# Rubella-associated granulomatous inflammation presenting as subcutaneous nodules in the lower extremities

**DOI:** 10.1016/j.jdcr.2025.12.034

**Published:** 2025-12-29

**Authors:** Amritpal Kooner, Alexandra Stroia, Jarett Anderson, David Fivenson, Michael DeWall, Karolyn A. Wanat

**Affiliations:** a Midwestern University, Downers Grove, Illinois; b Department of Dermatology, Trinity Health Ann Arbor, Ypsilanti, Michigan; c Department of Dermatology and Pathology, Medical College of Wisconsin, Milwaukee, Wisconsin

**Keywords:** granulomas, JAK, rubella virus, viral granuloma

## Introduction

Rubella virus (RuV) is an enveloped positive-sense single-stranded RNA virus belonging to the Togaviridae family. Since the introduction of the live attenuated RuV vaccine as part of the measles, mumps, and rubella series, the incidence of RuV and congenital RuV syndrome worldwide has significantly reduced.^1^ Recently, RuV, both vaccine-derived and wild-type, has been shown to be associated with granulomatous inflammation in pediatric and adult patients with inborn errors of immunity,^2^, ^3^, ^4^, ^5^, ^6^ and even in clinically immunocompetent patients.^1^^,^^7^

## Case presentation

**Fig 1:**
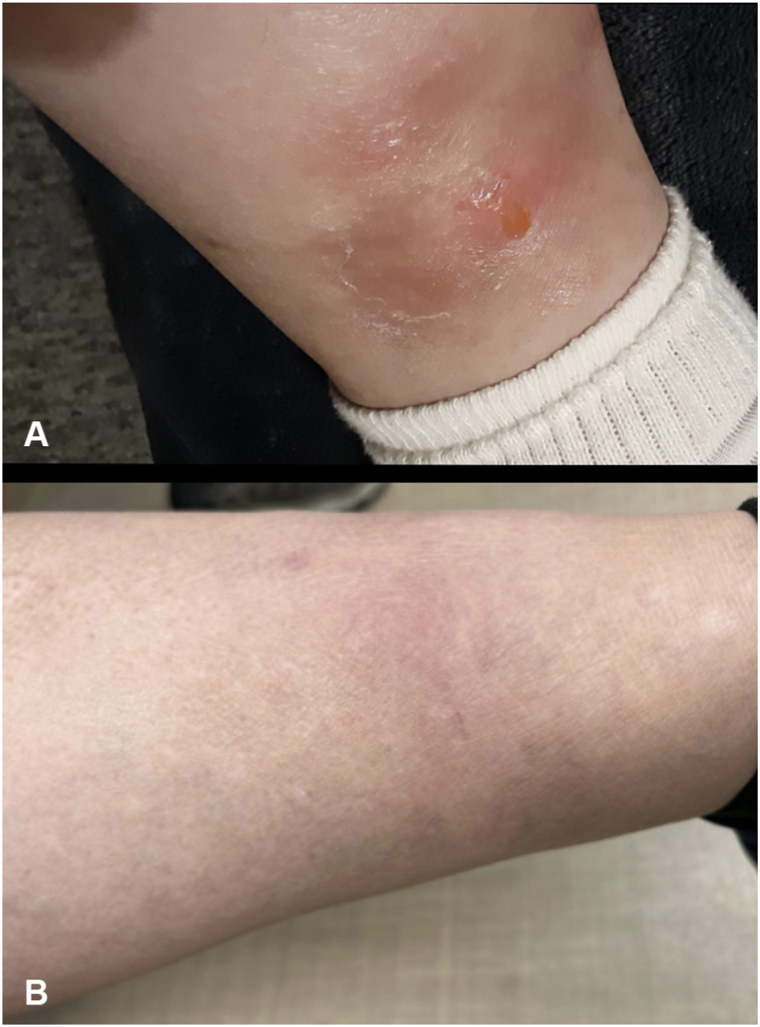
Lower extremity subcutaneous nodules before and after treatment. Clinical appearance of pink subcutaneous nodules on lower extremities with scant drainage of clear fluid at initial presentation **(A)** and resolution of nodules after treatment with upadacitinib 15 mg daily **(B)**.

**Fig 2:**
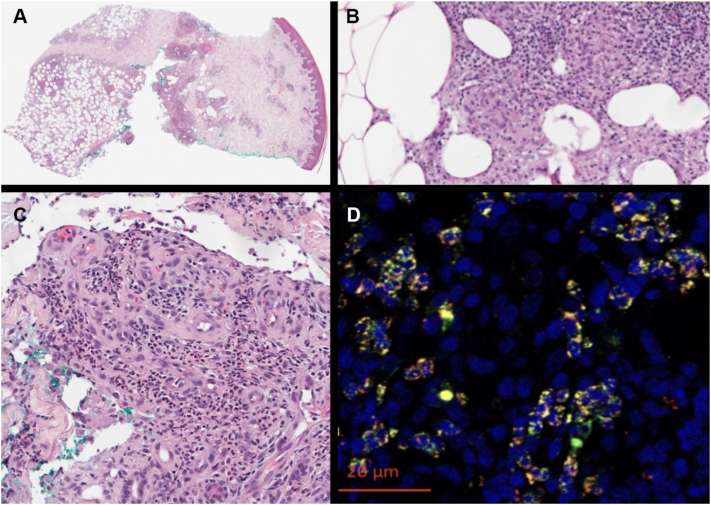
Histopathology of right lower leg granuloma biopsy demonstrating inflammation confined to subcutaneous fat with intralobular inflammation and mild septal thickening. (**A,** original magnification 4×) Granulomatous and dense lymphocytic infiltrate without lymphocytic rimming (**B,** 10× original magnification) and neutrophilic predominance in deep dermis (**C,** 20× original magnification). **D,** Immunohistochemical staining for rubella virus capsid antigen (red) demonstrates strong staining localizing to neutrophils (green; myeloperoxidase and blue); DAPI (4′,6-diamidino-2-phenylindole) highlighting nuclei. Monoclonal antibodies used included anti–rubella virus capsid (Centers for Disease Control and Prevention, Division of Viral Diseases), antimyeloperoxidase, and anti–CD206 antibodies.

A 47-year-old woman with no underlying medical comorbidities and no history of birth, residence, or recent travel outside the United States presented with a 5-year history of recurrent, cyclic episodes of tender, draining subcutaneous nodules chiefly involving the lower extremities (Fig 1, *A*). On physical examination, she had pink, tender nodules on the lower legs with minimal clear fluid drainage. Histopathology demonstrated a brisk, deep dermal and prominent subcutaneous infiltrate (Fig 2, *A*), characterized by a septal-lobular panniculitis with widened septae and fat lobules densely infiltrated by lymphocytes and histiocytes and multinucleated giant cells forming granulomas. Complete blood count was notable for a mildly elevated relative monocyte count (10.2%), with all other parameters within normal limits. Screening for human immunodeficiency virus, hepatitis, and tuberculosis was negative. She was up to date on her vaccinations, including the measles, mumps, and rubella vaccines as a child. She underwent empiric treatment for a presumed diagnosis of erythema nodosum with 600 mg daily of saturated solution of potassium iodide, hydroxychloroquine 200 mg twice daily, and multiple rounds of 20 mg/cc intralesional triamcinolone acetonide, all of which yielded only partial improvement. Given a lack of therapeutic response, a repeat right lower leg biopsy was performed, which also demonstrated lobular granulomatous panniculitis with mild septal thickening, and associated lymphocytes but an absence of atypical cells and lymphocytic rimming (Fig 2, *B* and *C*). Periodic acid–Schiff and Fite stains were negative. Due to the continued concern for infectious etiology, this specimen was tested for RuV (Centers for Disease Control and Prevention).

Double immunofluorescent staining of biopsy specimens using a RuV capsid antibody showed strong nuclear RuV capsid antigen staining (Fig 2, *D*). Reverse transcriptase-polymerase chain reaction for RuV on testing on formalin-fixed tissue, urine, and nasopharyngeal swabs was negative using standard protocols; however, given the technical limitations of RNA detection in fixed tissue, a negative result does not exclude infection. Rubella serology showed negative immunoglobulin M and positive immunoglobulin G at 50 IU, representative of her immunized status. Genotyping to distinguish wild-type from vaccine-derived RuV was inconclusive.

The patient was started on a combination therapy regimen consisting of hydroxychloroquine 600 mg 5 days per week and 400 mg on the remaining 2 days, doxycycline 100 mg twice daily, and upadacitinib 15 mg daily. This regimen led to significant symptom control and reduction in lesion frequency. However, discontinuation of upadacitinib resulted in recurrence of nodules, which subsequently resolved upon resumption (Fig 1, *B*).

## Discussion

Rubella virus–associated granulomatous disease (RuV-GD) has been infrequently reported in adult immunocompetent individuals. Although initially recognized in immunocompromised individuals, particularly those with primary immunodeficiencies harboring persistent vaccine-derived RuV, more recent reports have identified RuV-GD in immunocompetent adults,^1^^,^^2^^,^^7^ suggesting that RuV can persist in the skin irrespective of immune status. Given its rarity, recent characterization, and reliance on specialized testing, RuV-GD may be underdiagnosed.

Histopathologically, RuV-GD in immunocompetent adults has been typically characterized by dermal granulomatous inflammation and may mimic sarcoidosis, CD8+ granulomatous cutaneous T-cell lymphoma, or labeled as idiopathic granulomas.^1^^,^^7^ Caseation or necrosis is often evident, and workup for an infectious etiology is typically negative.^7^ This case is unique in that it expands the presentation of RuV-GDs to include panniculitis. Diagnosis relies on immunohistochemistry for RuV capsid protein, reverse transcriptase-polymerase chain reaction, and genomic sequencing.^1^ In this case, RuV antigen was found to co-localize within the neutrophils and not macrophages, although the inverse has been observed in other cases.^4^^,^^5^

Potential transmission of both wild-type and vaccine-derived RuV from patients with granulomatous lesions has not been well studied. Testing of serology, urine, and nasopharyngeal and oropharyngeal swabs is recommended in patients with suspected or proven RuV-GD.^2^ In one reported instance, a patient with RuV granulomas shed live, replication-competent vaccine-derived RuV in respiratory secretions and urine for 1.5 years after diagnosis.^3^ However, no secondary transmission was found in testing of close contacts. Although secondary spread has not been documented, those with confirmed shedding of replication-competent virus should be advised to avoid close contact with pregnant and immunocompromised individuals.^7^

Given the reported efficacy of janus kinase inhibitors in various granulomatous conditions,^8^ our patient’s response to upadacitinib 15 mg daily was not entirely unexpected. She experienced significant clinical improvement in therapy with demonstration of clinical response related to treatment discontinuation and reinitiation. Involvement of the JAK/STAT signaling pathway, particularly through inhibition of proinflammatory cytokines such as interleukin-6, interferon-gamma, and tumor necrosis factor alpha, may be crucial to granuloma formation in RuV-GD.^8^ Efficacy of JAK inhibitors may stem from its ability to suppress cytokine-driven inflammation and immune cell recruitment, thereby interrupting the chronic granulomatous process associated with RuV persistence.^9^ Given the limited success of previously reported treatments, upadacitinib represents a novel therapeutic option in RuV-GD. While this outcome is promising, further research is needed to evaluate the long-term safety, efficacy, and broader applicability of JAK inhibitors in the management of RuV-GD.

## Conclusion

This case highlights an uncommon clinicopathologic presentation of RuV-GD characterized by panniculitic inflammation in an immunocompetent adult. It broadens the current understanding of RuV-GD, emphasizing the variability in both clinical and histologic manifestations. The patient’s response to upadacitinib supports its potential role as a novel and effective treatment for RuV-GD. This outcome underscores the need for further research into JAK inhibitors as targeted therapies in chronic granulomatous conditions linked to persistent viral infections.

## Conflicts of interest

None disclosed.
